# Draft genome sequence of *Streptomyces* sp. MNU76, isolated from Indus River sediment, reveals uncharacterized biosynthetic gene clusters

**DOI:** 10.1128/mra.01362-25

**Published:** 2026-04-15

**Authors:** Aruna Kumari, Garima Suneja, Sonam Nain, Saraswati Awasthi, Meenakshi Sharma, Rakesh Sharma

**Affiliations:** 1 Genomics and Genome Biology, CSIR-Institute of Genomics and Integrative Biology, Council of Scientific and Industrial Research (CSIR), New Delhi, India; 2 Academy of Scientific and Innovative Research (AcSIR) 550336, Ghaziabad, India; University of Strathclyde, Glasgow, United Kingdom

**Keywords:** genome analysis, *Streptomyces*, biosynthetic gene clusters, antifungal

## Abstract

Here, we present the draft genome sequence of *Streptomyces* sp. MNU76 isolated from river sediment. The genome comprises 37 contigs, 10.5 Mb in size, and 71% GC content. The species harbors multiple predicted biosynthetic gene clusters, including several polyketide synthase pathways; the natural products they encode remain uncharacterized.

## ANNOUNCEMENT

*Streptomyces* are Gram-positive bacteria found in soil, water, and sediment, and in symbiotic relationships (1), and are known to produce numerous natural products with diverse bioactivities. Freshwater ecosystems represent a promising source of bioactive compounds with potential clinical applications (2).

**Fig 1:**
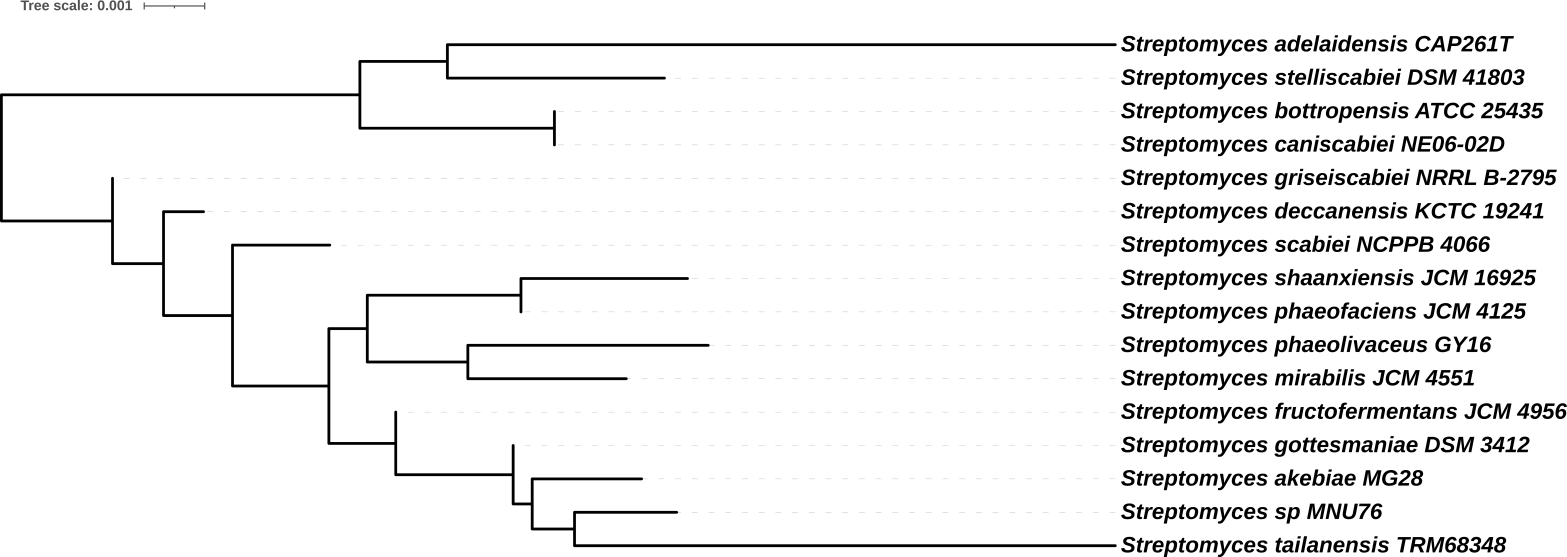
16S rRNA gene-based phylogenetic tree of *Streptomyces* sp. MNU76, showing the phylogenetic position of strain MNU76 with other *Streptomyces* species. The tree was generated using the TYGS server employing the genome BLAST distance phylogeny (GBDP) method on 16S rRNA sequences. The tip labels show the strain names.

We report the draft genome sequence of *Streptomyces* sp. strain MNU76 isolated from 100 g of Indus River sediment (1 g used for plating) collected at a depth of 10 cm in Leh, India (34°03′41.3″ N, 77°38′21.9″ E). The sample was homogenized, spread on actinomycete isolation agar, and incubated at 30°C for 6 days. The *Streptomyces* colonies were picked, grown on tryptic soy agar (pH 7.2), and incubated at 30°C for 5 days to obtain pure colonies. The genomic DNA was extracted using the DNeasy UltraClean Microbial Kit (Qiagen). The library was prepared using a Nextera XT DNA Library kit and sequenced with 2 × 300 bp reads on the MiSeq (Illumina). Paired-end raw read pairs (8,330,624) were quality-checked with FastQC v0.11.9 and trimmed using Trimmomatic v0.38 (3). The same DNA was used without size selection for library preparation with the ligation sequencing kit SQK-LSK114 (Oxford Nanopore Technologies) and sequenced on an FLO-MIN106 R9 flow cell using a MinION Mk1 (HAC base calling), yielding 213,449 Nanopore reads (mean length, 1.5 kb; N50, 2.0 kb). Trimming and adapter removal were done with Porechop v0.2.4 (4). Nanopore reads were assembled using Canu v1.8 (5) and polished with MiSeq reads via Pilon v1.8 (6). Genome completeness was assessed using checkM v1.1.3 using lineage_wf workflow (7). Phylogeny was reconstructed using TYGS v403 (8) based on the genome BLAST Distance Phylogeny method. ANI was calculated using FastANI v1.33. Genome annotation was performed using PGAP v6.8 (RefSeq) from NCBI (9). All tools were run using the default parameters. The draft genome of *Streptomyces* sp. MNU76 has a genome size of 10.5 Mb, a GC content of 71%, and a genome completeness of 99.6%. The hybrid genome assembly produced 37 contigs with an N50 value of 554 kb and genome coverage of 124×. The 16S rRNA-based phylogenetic tree indicated that *Streptomyces* sp. MNU76 is related to *Streptomyces tailanensis* TRM68348 (GCF_008386495.1), *Streptomyces akebiae* MG28 (GCF_019599145.1), and *Streptomyces gottesmaniae* DSM 3412 (GCF_031845845.1) (Fig. 1). Average nucleotide identity (ANI) comparisons with these strains yielded values of 87.6%, 92.4%, and 92%, respectively, indicating that *Streptomyces* sp. MNU76 might be a novel species. It contains 9,602 genes, comprising 8,578 coding sequences (CDSs), 72 transfer RNAs (tRNAs), 17 ribosomal RNAs (rRNAs), and three non-coding RNAs (ncRNAs) (NCBI RefSeq assembly GCF_020982545.1). The antiSMASH v8.0.4 (10) analysis predicted 42 biosynthetic gene clusters (BGCs) in the genome of *Streptomyces* sp. MNU76; full, contig-resolved annotations are available via Zenodo (DOI: https://doi.org/10.5281/zenodo.18477189). It harbors several uncharacterized BGCs, including multiple predicted PKS-type pathways that show no similarity to known gene clusters. The diversity and richness of these clusters highlight the untapped metabolic potential of *Streptomyces* sp. MNU76. It contains several predicted PKS and NRPS-like clusters that potentially encode pharmacologically active compounds yet to be explored.

## Data Availability

The whole-genome shotgun project has been deposited in the National Center for Biotechnology Information (NCBI) in GenBank under accession number GCF_020982545.1. The data are available under NCBI Bioproject PRJNA267646. The raw reads have been deposited in NCBI’s SRA database under accession numbers SRR8841084 (Nanopore reads) and SRR8841085 (Illumina reads). The antiSMASH annotations were uploaded to the Zenodo repository as a data set with https://doi.org/10.5281/zenodo.18477189.
